# Carcinome urothélial sur un calice rénal exclu révélé par une métastase cérébrale

**DOI:** 10.11604/pamj.2019.34.184.15767

**Published:** 2019-12-06

**Authors:** Ahsaini Mustapha, Bienvenu Bega Shamalirwa, Jean Paul Omana Wembonyama, Richepin Tidahy, Efared Boubacar, Hind El Fatemi, Mellas Soufiane, Tazi Mohammed Fadl

**Affiliations:** 1 Service d'Urologie, CHU Hassan II of Fèz, Fèz, Morocco; 2 Service d'Anatomo-pathologie CHU Hassan II de Fèz, Fèz, Maroc

**Keywords:** Carcinome urothélial, diagnostic, métastases cérébrales, tumeurs kystiques du rein, Urothelial carcinoma, diagnosis, brain metastasis, cystic tumors of the kidney

## Abstract

Le carcinome urothélial est caractérisé par une symptomatologie bruyante faite d'hématurie macroscopique généralement. Nous rapportons ici le cas d'une patiente sans antécédent de tabagisme ni de carcinome urothélial connu; ce qui en fait à notre connaissance un cas unique et une première où le carcinome urothélial se révèle par une métastase cérébrale.

## Abstract (en)

Urothelial carcinoma is usually characterized by severe symptoms including macroscopic haematuria. We here report the case of a female patient without a history of smoking or urothelial carcinoma, making it, to our knowledge, a unique case and a first case of urothelial carcinoma revealed by brain metastasis.

## Introduction

Le carcinome urothélial est caractérisé par une symptomatologie trop bruyante, la majorité des cas par la survenue d'une hématurie caillotante intermittente macroscopique d'une part et d'autre part par la survenue des récidives tumorales, la progression de la maladie et des métastases. Les métastases cérébrales des cancers urothéliaux sont peu fréquentes et rarement isolées survenant dans un contexte de multi métastases [1]. La survenue d'un carcinome urothélial des voies excrétrices dans un calice rénal qui se révèle par une métastase au niveau cérébral est inhabituelle (< 1%) [1].

## Patient et observation

**Figure 1:**
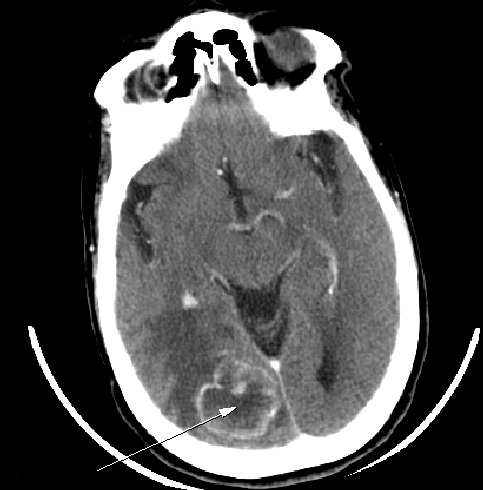
Coupe axiale montrant la lésion cérébrale au niveau occipital droit

**Figure 2:**
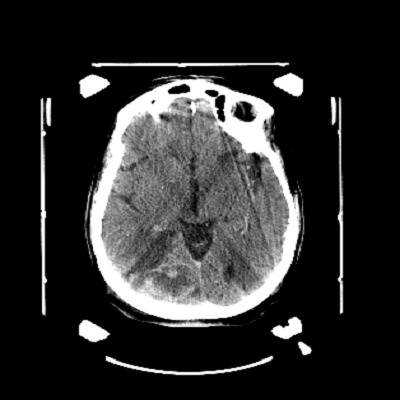
Coupe axiale de la lésion cérébrale lors de la biopsie stéréotaxique

**Figure 3:**
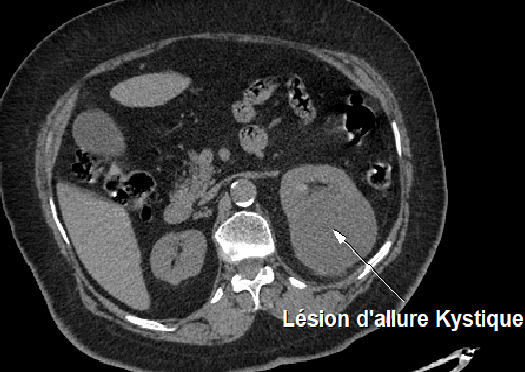
Scanner abdomino-pelvien en coupe axiale montrant la lésion d’allure kystique calcifiée au niveau du pôle supérieur du rein gauche

**Figure 4:**
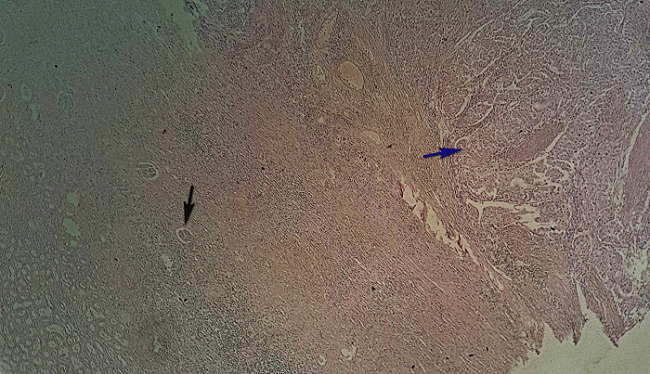
Coupe histologique de la lésion rénale gauche au grossissement X50

**Figure 5:**
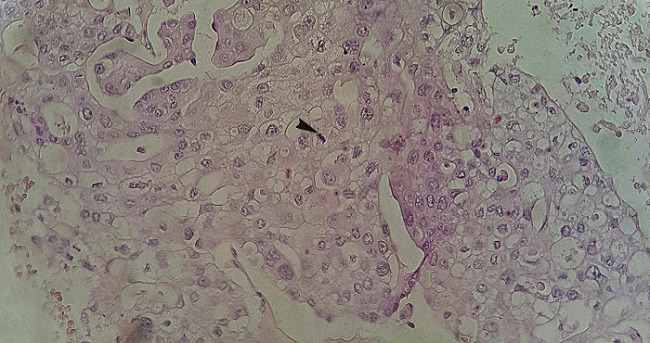
Coupe histologique de la tumeur au grossissement X200

Nous rapportons ici le cas d'une patiente de 72 ans suivie en cardiologie pour une cardiopathie hypertensive et ischémique connue et traitée sans notion de tabagisme qui a été admise en hospitalisation en neurochirurgie pour des céphalées persistantes d'intensité croissante associées à des troubles de la vue chez qui le scanner cérébral (Figure 1) réalisé avait conclu à deux lésions cérébrales pariétale droit et occipital homolatéral qui évoquait en premier des localisations secondaires d'une pathologie tumorale à rechercher et la découverte d’une lésion rénale gauche ayant motivé une demande ultérieure d'un avis dans notre service d'urologie. Une biopsie stéréotaxique cérébrale (Figure 2) avait été pratiquée dont la réponse des analyses anatomo-pathologiques est revenue en faveur d'une métastase cérébrale d'un carcinome urothélial. La patiente sera transférée dans notre service où un uro-tomodensitometire sera réalisé et va mettre en évidence une lésion d'allure kystique calcifiée au niveau du pôle supérieur du rein gauche (Figure 3) constituant ainsi l'indication d'une néphrectomie gauche totale élargie. Les analyses de la pièce opératoire sont revenues en faveur d'un carcinome urothélial papillaire de grade 3 et de haut grade infiltrant les cavités calicielles supérieures du rein gauche avec une différenciation malpighienne estimée à 5% (Figure 4, Figure 5). La patiente a bénéficié d'une chimiothérapie adjuvante à base de Gemcitabine avec une bonne évolution clinique sans une récidive tumorale ou métastatique avec un recul de 15 mois.

## Discussion

A notre connaissance, notre patiente constitue le deuxième cas décrit [2] de métastases vasculaires au cerveau sans antécédents de chimiothérapie et en plus pour la première fois sans antécédent de carcinome urothélial connu, ce qui en fait un cas clinique rare. Les signes neurologiques ont constitué la manifestation inaugurale chez notre patiente contrairement dans la pratique courante où on retrouve soit une hématurie macroscopique indolore soient des douleurs lombaires [3]. Dans l'évolution d'un carcinome urothélial des voies excrétrices supérieures; la métastase par greffe de cellules tumorales au niveau de la vessie ou par contiguïté étant le mode le plus répandue, justifiant la néphro-urétérectomie totale avec collerette vésicale [4], on pouvait s'attendre à une récidive sur le moignon urétéral distal ou carrément à une récidive vésicale mais notre patiente a présenté au cours de l'évolution de sa maladie des métastases ganglionnaires médiastinales et pulmonaires traité par chimiothérapie (gemcitabine) et la radiothérapie pour les métastases cérébrales. La localisation d'un carcinome urothélial des voies excrétrices dans un calice supérieur exclu est rare privilégiant l'hypothèse diagnostique piège d'une tumeur kystique rénale (épaississement de la paroi kystique et présence des calcifications intra-kystiques) beaucoup plus habituelle dans notre cas, seul l'examen anatomo-pathologique a pu trancher qu'il s'agissait bien d'un carcinome urothélial des voies excrétrices supérieures, le diagnostic d'emblée difficile en imagerie à moins d'un signe indirect sous forme d'hydronéphrose sans obstacle visible ou bien d'un néphrogramme amputé. Pour notre patiente, il s'est posé plutôt sous forme d'une dilatation kystique calcifié (calice exclu).

## Conclusion

La découverte d'un carcinome urothélial par une métastase cérébrale est rare d'une part et d'autre part la localisation d'un carcinome urothélial des voies excrétrices dans un calice exclu simulant une lésion kystique calcifié est inhabituelle rendant cette combinaison exceptionnelle. A notre connaissance, c'est le premier cas décrit d'une métastase cérébrale révélant un carcinome urothélial chez une patiente sans antécédents de tumeur de vessie ou de tumeur de la voie excrétrice supérieure.

## Conflits d’intérêts

Les auteurs ne déclarent aucun conflit d'intérêts.
