# Corrigendum: Carcinome urothélial sur un calice rénal exclu révélé par une métastase cérébrale

**DOI:** 10.11604/pamj.2020.36.50.23908

**Published:** 2020-06-01

**Authors:** The Pan African Medical Journal

**Affiliations:** 1 The Pan African Medical Journal, Nairobi, Kenya

**Keywords:** Corrigendum, carcinome urothélial, diagnostic, métastases cérébrales, tumeurs kystiques du rein, Corrigendum, urothelial carcinoma, diagnosis, brain metastasis, cystic tumors of the kidney

## Abstract

Ce Corrigendum vise à rectifier l’oubli d’un nom d’auteur dans la liste d'auteurs de l’article original « Carcinome urothélial sur un calice rénal exclu révélé par une métastase cérébrale ». Pan Afr Med J. 2019;34:184.

## Abstract (en)

This Corrigendum modifies the original article in which an author’s name was missing: "Carcinome urothélial sur un calice rénal exclu révélé par une métastase cérébrale". Pan Afr Med J. 2019;34:184.

## Corrigendum

Le Dr Farih Moulay Hassan n’avait pas été ajouté dans la première liste d’auteur de l’article original [1]. Pourtant, il devait y figurer en tant que dernier auteur de la liste, son affiliation étant “Service d'Urologie, CHU Hassan II, Fèz, Maroc”. Il a contribué au même titre que les autres auteurs dans cet article.

La citation correcte est: Ahsaini Mustapha, Bienvenu Bega Shamalirwa, Jean Paul Omana Wembonyama, Richepin Tidahy, Efared Boubacar, Hind El Fatemi, Mellas Soufiane, Tazi Mohammed Fadl, Farih Moulay Hassan. Carcinome urothélial sur un calice rénal exclu révélé par une métastase cérébrale. Pan Afr Med J. 2019;34:184. doi: 10.11604/pamj.2019.34.184.15767.
